# Multidrug-Resistant *Escherichia coli* in Broiler and Indigenous Farm Environments in Klang Valley, Malaysia

**DOI:** 10.3390/antibiotics14030246

**Published:** 2025-02-28

**Authors:** Yuvaneswary Veloo, Syahidiah Syed Abu Thahir, Rafiza Shaharudin, Sakshaleni Rajendiran

**Affiliations:** Environmental Health Research Centre, Institute for Medical Research, National Institutes of Health, Ministry of Health, Shah Alam 40170, Malaysia; syahidiah@moh.gov.my (S.S.A.T.); sakshaleni@moh.gov.my (S.R.)

**Keywords:** antimicrobial resistance, multidrug resistance, *Escherichia coli*, chicken, environment

## Abstract

**Background/Objectives:** The global health threat posed by antimicrobial resistance (AMR) is a cause for serious concern. Poultry farming in Asia, particularly with indiscriminate use, has been identified as a major contributor to AMR, resulting in the emergence of multidrug-resistant (MDR) bacteria, particularly *Escherichia coli* (*E. coli*). Considering the environment’s role in harboring pathogens, this study aimed to determine the distribution of MDR *E. coli* in the environments of broiler and indigenous farms in Klang Valley, Malaysia. **Methods:** Soil and effluent samples were collected from 30 poultry (19 broiler and 11 indigenous) farms. Selective chromogenic *E. coli* agar plates were used for the isolation of bacteria. The VITEX^®^ 2 system was employed for identification and susceptibility testing. **Results:** A total of 117 *E. coli* isolates were recovered. The isolates were highly resistant to ampicillin (76.1%), followed by trimethoprim-sulfamethoxazole (47.9%), and ampicillin-sulbactam (46.2%). AMR rates were higher in broiler farms (91.3%) than in indigenous farms (64.6%) (*p* < 0.05). The high multiple antibiotic resistance index in the environment of broiler farms (0.22) implies a higher risk of contamination compared to indigenous farms (0.10). **Conclusions:** The elevated levels of AMR observed in broiler farms underscore the need for collaborative efforts across sectors to address this issue. As AMR is a global One Health issue, monitoring AMR in the environment is essential to complement existing health programs. Implementing best practices, biosecurity, hygiene, continuous monitoring, and food safety management systems is crucial to reduce antimicrobial use and curb the rise of MDR bacteria.

## 1. Introduction

In the era of fast-food chains and rapid urbanization, there is growing demand for poultry in food consumption. Consequently, farmers must acquire multiple ways of breeding and raising poultry for its meat and egg products [1]. One popular practice is broiler breeding, as it can produce on a large scale with a higher yield [2], in contrast to indigenous (free-range) chicken breeding, which can be time-consuming and challenging, as it is exposed to more diseases [3]. Both have their sets of challenges. Since broilers are grown on a large scale, these farms are usually densely populated, which makes it challenging to maintain sanitation [4]. Consequently, broilers are prone to various bacterial infections if not managed properly [5]. Thus, antibiotics are used to prevent disease to achieve a higher poultry production rate. On top of that, the environmental impact of broiler practices, in which waste management, if not maintained adequately, can leach harmful bacteria into surrounding ecosystems, poses significant risks to both human health and wildlife [6]. 

*Escherichia coli* is one of the pathogenic bacteria found in poultry farms that can lead to disease in the chicken and manifest as severe loss to the farmers. As such, antibiotics are usually used as therapeutics and prophylactics to manage the spread of infection [7]. This practice can often lead to multi-drug resistance (MDR) in *E. coli*, as continuous exposure to antimicrobials results in selective pressure and the mutated bacteria remain despite the presence of antimicrobial drugs [8]. The prevalence of MDR *E. coli* in poultry and the environment reported by Lawal et al. was in the range of 14.8 to 100% in Malaysia [9]. Many countries have initiated bans on antimicrobial use in poultry as growth promoters; similarly, antibiotics have been classified as controlled substances in Malaysia by the Ministry of Health (NPRA) through the Poison Act 1952 [10], while the Department of Veterinary Services controls the use of antibiotics in feed or for prophylaxis [11]. Some of the antibiotics that have been listed as prohibited to use in animal feed are colistin, erythromycin, enrofloxacin, tetracycline, ceftiofur, tylosin, and fosfomycin [12].

In Malaysia, farming practices are widely dispersed. Major cities like Kuala Lumpur and the bordering state of Selangor are classified as the Klang Valley region, with exponential growth both in population and infrastructure; thus, demand for poultry farming has been growing [13,14]. The poultry consumption per capita was 49.7 kg per person in 2021, and it is forecast to reach 51.28 kg per capita by 2025. The increasing trend has made Malaysia among the top meat consumers worldwide [15].

Due to the demand and growing need for poultry chickens, there is a need to monitor the antimicrobial resistance trends of broiler and indigenous farms in the Klang Valley region, as poultry waste containing *E. coli* can contaminate soil and water sources, leading to major environmental impacts. While previous studies highlighted different bacterial species [16,17], no study has been conducted on the distribution of *E. coli* in broiler and indigenous farms. Thus, this study aims to determine the distribution of antimicrobial resistance (AMR) and MDR *E. coli* in the environments of broiler and indigenous farms.

## 2. Results

### 2.1. AMR of E. coli in the Poultry Farm Environment

**Figure 1:**
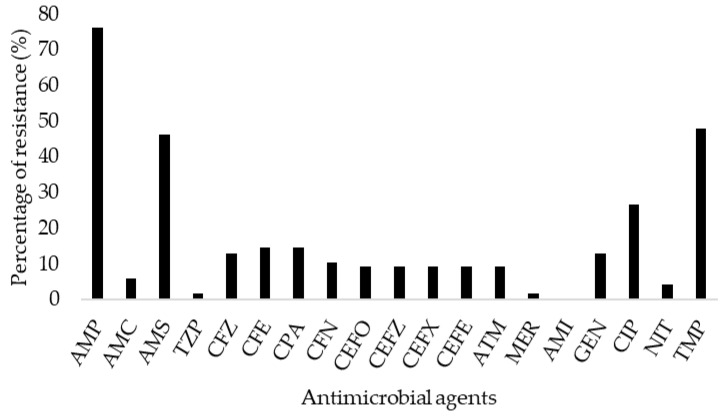
AMR percentage rate of *E. coli* in the poultry farm environment. Abbreviations: AMP, ampicillin; AMC, amoxicillin-clavulanic acid; AMS, ampicillin-sulbactam; TZP, piperacillin-tazobactam; CFZ, cefazolin; CFE, cefuroxime; CPA, cefuroxime axetil; CFN, cefoxitin; CEFO, cefotaxime; CEFZ, ceftazidime; CEFX, ceftriaxone; CEFE, cefepime; ATM, aztreonam; MER, meropenem; AMI, amikacin; GEN, gentamicin; CIP, ciprofloxacin; NIT, nitrofurantoin; TMP, trimethoprim-sulfamethoxazole.

A total of 117 *E. coli* isolates were recovered from the environmental samples and these isolates were included in the present study for analysis of antimicrobial resistance. The prevalence of *E. coli* in both broiler and indigenous farm environment samples is shown in Table S1. Based on the Chi-squared test, there was no significant difference in resistance towards the selected antimicrobials among the isolates recovered from soil and effluent isolates (*p*-value = 0.4). Therefore, the samples were further analyzed as overall environmental samples. The highest AMR rate was recorded by ampicillin (76.1%, 89/117 isolates), followed by trimethoprim-sulfamethoxazole (47.9%, 56/117 isolates) and ampicillin-sulbactam (46.2%, 54/117 isolates) (Figure 1).

The AMR profiling of the isolates based on the number of antimicrobial categories is shown in Table 1, with a total of 53 MDR isolates (45.3%), with 19 antimicrobials representing 11 different antimicrobial categories. Of those, the penicillin category was detected in 100%, followed by penicillin plus β-lactamase inhibitors and the sulfonamide class, with 75% each. Two isolates demonstrated resistance to eight antimicrobial categories, while six isolates exhibited resistance to seven antimicrobial categories. Furthermore, 11 isolates were resistant to ampicillin and third- and fourth-generation cephalosporins, labeled as extended-spectrum β-lactamase (ESBL)-producing bacteria. Additionally, two of the isolates were resistant to meropenem, the carbapenem group. 

### 2.2. AMR Pattern and MDR E. coli in Broiler and Indigenous Farm Environments

**Figure 2:**
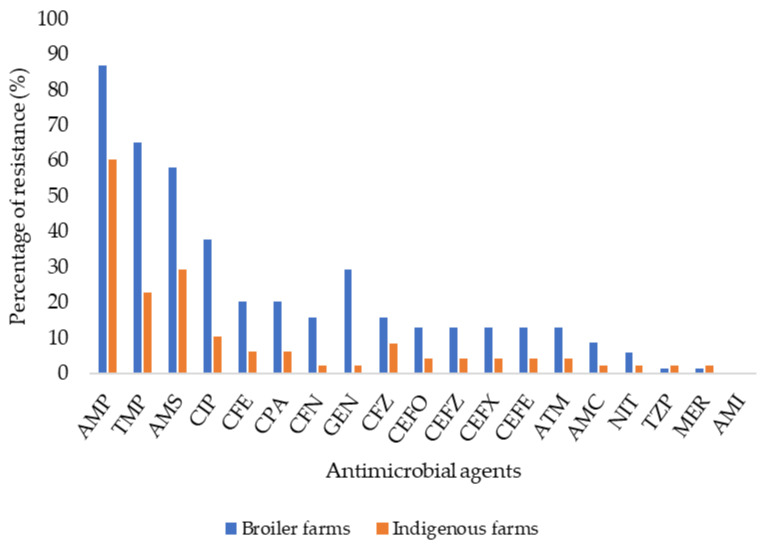
AMR percentage rate based on the type of farm. Abbreviations: AMP, ampicillin; TMP, trimethoprim-sulfamethoxazole; AMS, ampicillin-sulbactam; CIP, ciprofloxacin; CFE, cefuroxime; CPA, cefuroxime axetil; CFN, cefoxitin; GEN, gentamicin; CFZ, cefazolin; CEFO, cefotaxime; CEFZ, ceftazidime; CEFX, ceftriaxone; CEFE, cefepime; ATM, aztreonam; AMC, amoxicillin-clavulanic acid; NIT, nitrofurantoin; TZP, piperacillin-tazobactam; MER, meropenem; AMI, amikacin.

Among the 30 poultry farms, 69 *E. coli* isolates were successfully recovered from the environmental samples of 19 broiler farms, and 48 *E. coli* isolates were obtained from environmental samples of 11 indigenous farms. A higher resistance rate was observed in the isolates from broiler farms (91.3%, 63/69 isolates) compared to those from indigenous farms (64.6%, 31/48 isolates). As shown in Figure 2, resistance to all the antimicrobial agents was higher in *E. coli* isolates from broiler farms, except for piperacillin-tazobactam and meropenem, where only one isolate was found for each. Since the data did not follow a normal distribution based on the Kolmogorov–Smirnov test, the Mann–Whitney U test was used for all the analyses. The resistance rate was significantly higher in broiler farms compared to indigenous farms (63.8% vs. 25.0%, *p* < 0.0001, Mann–Whitney U test). Regarding MDR isolates, of the 53 isolates that were identified, 43 (84.3%) isolates were from broiler farms, while 10 (15.7%) isolates were from indigenous farms. 

### 2.3. MAR Index

**Figure 3:**
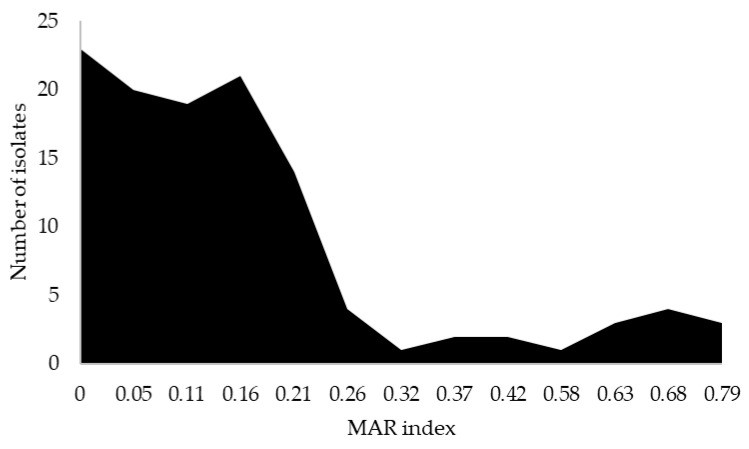
MAR index value in the overall poultry farm environment, with the number of *E. coli* isolates exceeding 0.20 indicating a high risk of AMR contamination.

**Figure 4:**
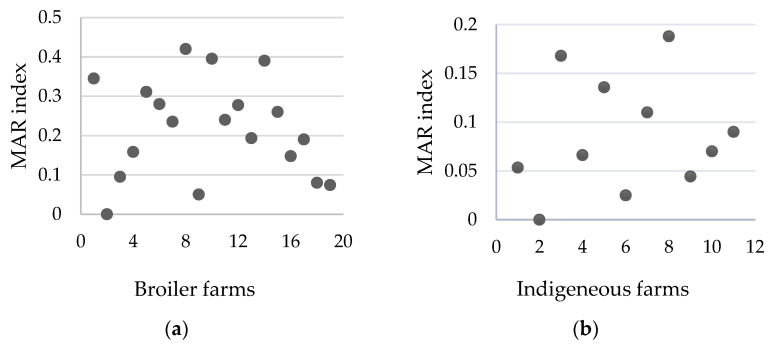
Distribution of *E. coli* MAR index based on farm type: (**a**) shows the MAR index of 19 broiler farms, with 10 farms recording an index of more than 0.2, indicating a high risk of contamination, and (**b**) shows the MAR index of 11 indigenous farms, with all recording an index of less than 0.2.

According to the MAR index percentage, 29.1% of *E. coli* isolated from the poultry farm environment were within the high-risk category. Figure 3 shows the number of isolates with MAR index values. There were 11 isolates that recorded a MAR index in the range of 0.58–0.79, with three isolates exhibiting the highest level of 0.79. Furthermore, the results showed a significant difference in the MAR index of *E. coli* isolates by farm type (*p* < 0.05). Based on the MAR index distribution, broiler farms exhibited a higher potential risk of contamination, with 10 out of 19 farms recording a MAR index of more than 0.2 compared to none in indigenous farms (Figure 4). 

## 3. Discussion

In this study, we investigated the distribution of AMR and MDR *E. coli* in the poultry farm environment, comparing broiler and indigenous farms. To the best of our knowledge, this is the first study in Malaysia to look into AMR profiles of *E. coli* in both broiler and indigenous farms. Our findings revealed a significantly higher resistance rate of *E. coli* isolates from broiler farm environments compared to indigenous farms. Specifically, the MDR rate was substantially higher in broiler farms (62.3%) compared to indigenous farms (20.8%). These align with previous studies, such as those by Hussain et al. and Rugumisa et al., which also found a significantly higher MDR rate in broiler farms than in indigenous farms [18,19]. Nevertheless, Hussain et al. only emphasized chicken meat samples and the MDR rate in broiler meat (68%; ceca 64%) and indigenous meat (8%; ceca 26%) [19] and Rugumisa et al. reported a significantly higher MDR frequency in commercial layer chickens than in indigenous chickens based on fecal samples [18]. Other studies comparing broiler and layer chickens also showed similar trends, such as in India, where the MDR rate of *E. coli* was 94% in broilers and 60% in layers, and in Bangladesh, where the MDR rate was 49.23% in broilers and 51.09% in layers based on the fecal samples [20,21]. 

The rapid emergence of MDR-resistant *E. coli* in broiler farms is reflected by the staggering demand for and production of chickens for food, increasing the potential for AMR development of pathogenic bacteria in poultry farms [22]. According to Hedman et al., who reviewed the AMR in poultry farming, the intensive nature of broiler farming can create environments conducive to the emergence and spread of AMR. The intensive practices and antibiotic use to promote growth and prevent diseases lead to increased AMR. On the other hand, indigenous farming also harbors AMR, but the lower use of antibiotics may help mitigate the development of resistance [2]. 

In this study, most of the *E. coli* isolates exhibited a high level of resistance to ampicillin and trimethoprim-sulfamethoxazole. Similarly, in Bangladesh, the trends observed in the poultry environment showed the highest resistance of *E. coli* isolates to ampicillin, ranging from 73.7% to 100%, and trimethoprim-sulfamethoxazole, ranging from 44.7% to 100% [23,24]. A study by Sebastian et al. also reported 100% resistance to ampicillin in *E. coli* isolates from the poultry environment [25]. Comparable resistance patterns were observed in other environmental studies conducted in poultry farms in Malaysia [22,26]. This widespread resistance is likely due to the long-term use of ampicillin in veterinary medicine, contributing to its diminished efficacy and increased resistance rates in both clinical and environmental settings. 

Regarding third- and fourth-generation cephalosporins in this study, resistance was predominantly observed in the broiler farm environment compared to indigenous farms. These third- and fourth-generation cephalosporins are listed as veterinary critically important antimicrobial agents (VCIA) and are not intended to be used as preventive treatment [27]. However, their use has been reported to control the mortality rate due to *E. coli* infections [28]. This could potentially drive the usage of these antimicrobials either in ovo or by injection, particularly in broiler production, which requires a high output. This practice may contribute to the increased AMR rates observed for these antimicrobials in broiler poultry farms [28,29]. In comparison to Ibrahim et al., who reported 7% resistance to cefotaxime in the poultry environment, in this study, the percentage of resistance to cefotaxime was higher in the broiler environment (13.0%) but lower in the indigenous environment (4.2%) [30]. 

However, the resistance rate to meropenem was detected as very low for both broiler and indigenous farm environments. This result agrees with studies from different countries. A study in Belgium and the Netherlands and another study in Bangladesh found no carbapenem-resistant *E. coli* in the poultry farms [24,31]. A study conducted in China on different origins of chicken showed a 4.9% resistance rate overall, with 1.9% resistance in layer farms, 48.7% in white-feather broiler farms, and all susceptible in live poultry markets [32]. Peng et al. reported a 2.3% resistance rate to meropenem in a surveillance study of AMR in *E. coli* in a pig farm [33]. This may be due to the reason that many countries, including the USA, do not approve the use of carbapenems in livestock based on the Animal Medicine Drug Use Clarification Act of 1994 and other similar regulations [34]. Meropenem is reserved as a last resort antibiotic in serious, multidrug-resistant infections in humans, as it is highly effective against a broad range of bacteria. 

The resistance patterns observed in this study align with findings from veterinary clinical isolates in the northern region of Peninsular Malaysia, which reported a 92.7% resistance rate to ampicillin [35]. A study by Ibrahim et al. on the east coast of Peninsular Malaysia found *E. coli* isolates from the chicken cloacal swab to be highly resistant to ampicillin (87.5%) and trimethoprim-sulfamethoxazole (83.3%) [36]. This pattern of resistance in poultry isolates across different regions of Malaysia is likely due to the widespread and prolonged use of these antimicrobials in the poultry industry, both for growth promotion and disease prevention. In terms of human health, the 2023 Malaysia National Antibiotic Resistance Surveillance report showed high resistance rates in clinical *E. coli* isolates from various hospitals in Malaysia, with resistance to ampicillin ranging from 61–64% and trimethoprim-sulfamethoxazole ranging from 31–36% between 2019 and 2023 [37]. Furthermore, the report indicated that the clinical isolates exhibited an average of 22.3% resistance to cefotaxime and 0.9% resistance to meropenem in the last 5 years [37]. The comparable findings in both poultry and clinical settings suggest potential transmission pathways between animals, humans, and the environment, highlighting the One Health implications of AMR. The low resistance to meropenem suggests that controlled and restricted usage for severe cases helps preserve their efficacy. 

The MAR index is a tool for assessing high-risk sources of contamination with antibiotics [38]. Based on the present findings, the environmental MAR index of *E. coli* isolated from broiler farms being significantly higher than indigenous farms implies that the environments of broiler farms are high-risk sources of contamination with antibiotics [39]. The higher resistance rates of antimicrobial agents and MAR indexes in broiler farm environments raise concerns about the possible usage of antimicrobials. Considering that the risk of disease transmission is high, especially due to overcrowding and stress, this factor could drive increased usage of antimicrobials to prevent illness and maintain the health and productivity of the flock [22,40]. Therefore, Hiroi et al. proposed to reduce or eliminate the usage of antimicrobials in poultry farms to reduce the AMR rate of particular antimicrobials. For example, removing ceftiofur from commercial broiler farms in Japan successfully reduced *E. coli* resistance to cephalosporin [41]. On that note, Korea decided to eliminate ceftiofur from the poultry industry in 2020 [42].

This study has some limitations. First, sampling was conducted in a specific region of Malaysia (the Klang Valley). This limits the findings to this particular region and not to the other parts of the country. Second, this study solely focused on environmental samples (soil and effluent) and did not include human, animal, and other sources of samples, such as feed, feces, and air, which limits the potential to correlate the three components of One Health. Also, this work relies on the culture-based method, which may underestimate the presence of resistance genes. These limitations are due to budget and time constraints. Therefore, future studies should consider wider sampling areas involving humans, animals, and the environment and using advanced techniques, such as metagenomic or molecular PCR. 

## 4. Materials and Methods

### 4.1. Environmental Sampling

A cross-sectional study was conducted from 2018–2019, focusing on the antimicrobial resistance of *E. coli*, a component of a broader study that was approved by the Ministry of Health, Malaysia with National Medical Research Register, NMRR-17-1198-36521 [17]. 

Using OneEpi version 3.01, assuming normality, the population size of the poultry environment, 1,000,000 with 80% power, a confidence level of 95%, and an unknown frequency of 50% for AMR in *E. coli*, the minimum number of isolates was determined to be 97. Using a simple random sampling method, environmental (99 soil and 51 effluent) samples were collected from 30 poultry farms. These farms were selected from the list of registered farms in Klang Valley that was provided by the Department of Veterinary Services, Selangor. The farm that was not registered under DVS, Selangor was excluded from this study. Besides that, layer farms were also excluded from this study, as our focus was only on broiler and indigenous farms. Approximately 25 g of soil samples were randomly collected from three different sites of the farm, focusing near the coop and the places where the chickens roam, using a metal spade. Meanwhile, 200 mL of effluent samples were collected upon availability in the drainage system or stagnant pool water. All the samples were then transported immediately to the laboratory via cool box.

### 4.2. Sample Collection, Preparation, and Isolation of E. coli

Once in the laboratory, all the samples were homogenized in the sterile plastic. From that plastic, 10 g of soil was added to 90 mL of peptone water. Similarly, 10 mL of effluent samples were added to 90 mL of peptone water. Subsequently, 10-fold dilution was performed by adding 1 mL of the aliquot into a new tube containing peptone water [17]. For isolation of bacteria from soil and effluent samples, 1.0 mL of samples from each dilution tube was pipetted onto the center of the commercially prepared chromogenic *E. coli* agar plate by a private accredited laboratory. The plates were then incubated aerobically at 37 °C for 24 h. Two to three representative blue color colonies, which are indicative of *E. coli,* were selected from plates containing 30 to 300 isolates for further purification via two times successive sub-culturing using Tryptic Soy agar to obtain pure colonies before proceeding with the identification and susceptibility testing.

### 4.3. Identification and Susceptibility Testing of E. coli

Similar to a recent paper, the VITEX^®^ 2 GN (bioMérieux, Nurtingen, Germany) was utilized for bacterial identification [16]. Concurrently, the AST-GN83 (bioMérieux, Nurtingen, Germany) susceptibility card was employed to determine the minimum inhibitory concentrations (MICs) of *E. coli* in accordance with the manufacturer’s guidelines. The AST-GN83 card contains 19 types of antimicrobials, including ampicillin, amoxicillin-clavulanic acid, ampicillin-sulbactam, piperacillin-tazobactam, cefazolin, cefuroxime, cefuroxime axetil, cefoxitin, cefotaxime, ceftazidime, ceftriaxone, cefepime, aztreonam, meropenem, amikacin, gentamicin, ciprofloxacin, nitrofurantoin, and trimethoprim-sulfamethoxazole. The details of the card can be found in the resource center of Biomerieux [43]. 

Approximately 3 mL of prepared 0.45% saline (bioMérieux) was dispensed into a pre-labeled, clear, 12 mm × 75 mm polystyrene test tube. The obtained pure *E. coli* colonies were inoculated into the saline-containing tube and mixed well until the turbidity reached 0.50 to 0.63 McFarland using a DensiCHEKTM Plus instrument (bioMérieux) [44,45]. The identification and testing results were interpreted using the Advanced Expert System™ (AES) software (https://www.biomerieux-microbio.com/tips-tricks-for-the-advanced-expert-system-aes/). The MIC analysis and the interpretation of antimicrobial susceptibility for *E. coli* were based on the Clinical Laboratory Standards Institute (CLSI) and the European Committee on Antimicrobial Susceptibility Testing (EUCAST) guidelines.

### 4.4. Statistical Analysis

All statistical analyses in this study were performed using the Statistical Package for Social Sciences (SPSS) software version 20 (IBM, New York, NY, USA). The AMR profiling for each antimicrobial was used to calculate the percentage of AMR in the poultry environment. The normality of the data was assessed using the Kolmogorov–Smirnov test, and it was found that the data were not normally distributed. Eventually, the farm groups were compared using the Mann–Whitney U test to assess whether there were significant differences between the two groups. The Chi-square test was used to compare the discrepancies between environmental samples (soil and effluent) in antimicrobial resistance. The results were considered significant when the *p*-value was less than 0.05.

### 4.5. Multiple Antibiotic Resistance (MAR) Index for Bacterial Isolate and Poultry Farm

The Multiple Antibiotic Resistance (MAR) index was calculated as the ratio of the number of antimicrobials to which an isolate was resistant to the total number of antimicrobials tested against the isolate. MAR index values of less than or equal to 0.2 indicate a low risk of antimicrobial-resistant bacteria contamination, while values > 0.2 indicate a high risk of antimicrobial-resistant bacteria contamination [46].

The MAR index, when applied to a single isolate, was calculated as follows:

MARindex = a/b(1)

a: The number of antibiotics to which each isolate was resistant;
b: The total number of antibiotics that were tested against an individual isolate.

The MAR index, when applied to a sample (farm), was calculated as follows:

MARindex = a/(b ⋅ c)(2)

a: The aggregate AMR score of all the isolates from the sample;
b: The number of antimicrobials that were tested;
c: The number of isolates from the sample.

## 5. Conclusions

This study highlights the concerning prevalence of resistant *E. coli* isolates, which were more prevalent in broiler poultry farm environments than indigenous poultry farms, exhibiting higher MDR rates and MDR indexes. These findings imply that broiler farms have the potential to serve as a reservoir area for AMR dissemination into the environment, potentially creating possibilities for pathways for the transmission of resistant bacteria between humans and animals via the environment. Therefore, it is imperative to monitor AMR in farm environments to complement the existing human and animal health program. Given that AMR is a global problem and regarded as a One Health issue, comprehensive management across sectors is essential. This includes adopting best practices in antimicrobial stewardship, biosecurity, and waste management to reduce antimicrobial use in the poultry industry. Further active measures, including strict hygiene practices, continuous monitoring and surveillance, and food safety management systems, are critical to restrain the rise of MDR bacteria. Further research should be considered targeting interventions in broiler farm environments to prevent transmission of AMR, aiming to safeguard human and animal health.

## Figures and Tables

**Table 1:** AMR profile of the MDR *E. coli* isolates.

Antimicrobial Resistance Profile	Number of Antimicrobial Categories	Isolates, n
AMP AMC AMS CFZ CFE CPA CFN CEFO CEFZ CEFX CEFE ATM TMP MER	8	2
AMP AMC AMS CFZ CFE CPA CFN CEFO CEFZ CEFX CEFE ATM TMP	7	3
AMP AMS CFZ CFE CPA CEFO CEFZ CEFX CEFE ATM CIP TMP	7	3
AMP CFZ CFE CPA CEFO CEFZ CEFX CEFE ATM GEN	5	2
AMP AMS CIP TMP	4	15
AMP AMS TMP	3	17
AMP CIP TMP	3	6
AMP AMS CIP	3	5

Abbreviations: AMP, ampicillin; AMC, amoxicillin-clavulanic acid; AMS, ampicillin-sulbactam; CFZ, cefazolin; CFE, cefuroxime; CPA, cefuroxime axetil, CFN, cefoxitin; CEFO, cefotaxime; CEFZ, ceftazidime; CEFX, ceftriaxone; CEFE, cefepime; ATM, aztreonam; GEN, gentamicin; CIP, ciprofloxacin; TMP, trimethoprim-sulfamethoxazole.

## Data Availability

The datasets generated and/or analyzed during the current study are available in the NIH-DaRS repository, or from the authors upon reasonable request.

## Supplementary Materials

The following supporting information can be downloaded at: https://www.mdpi.com/article/10.3390/antibiotics14030246/s1, Table S1: The details of the samples based on the type of the farm.
