# Supplementary material for: Multidrug-Resistant Escherichia coli in Broiler and Indigenous Farm Environments in Klang Valley, Malaysia
Source: Antibiotics (Basel). 2025 Feb 28;14(3):246. doi: 10.3390/antibiotics14030246 (PMC11939170; doi:10.3390/antibiotics14030246)
Supplement: Supplementary file 1 [file antibiotics-14-00246-s001.zip › antibiotics-3261433-supplementary.pdf]

Supplementary Table S1. The details of the samples based on the type of the farm

		Broiler	Indigenous
Number of farms		19	11
		Samples (no. of isolates)	Samples (no. of isolates)
Number of samples	Soil	57 (36)	33 (27)
	Effluent	27 (33)	24 (21)
	Total	84 (69)	57 (48)
Antimicrobial agents		No. of isolates (%)	No. of isolates (%)
Ampicillin	Soil	32 (88.9)	17 (63.0)
	Effluent	28 (84.8)	12 (57.1)
	Total	60 (87.0)	29 (60.4)
amoxicillin-clavulanic acid	Soil	1 (2.8)	1 (3.7)
	Effluent	5 (15.2)	1 (2.1)
	Total	6 (8.7)	0 (0)
ampicillin-sulbactam	Soil	22 (61.1)	5 (18.5)
	Effluent	18 (54.5)	9 (42.9)
	Total	40 (58.0)	14 (29.2)
piperacillin-tazobactam	Soil	1 (2.8)	1 (3.7)
	Effluent	0 (0)	0 (0)
	Total	1 (1.4)	1 (2.1)
cefazolin	Soil	5 (13.9)	2 (7.4)
	Effluent	6 (18.2)	2 (9.5)
	Total	11 (15.9)	4 (8.3)
cefuroxime	Soil	6 (16.7)	1 (3.7)
	Effluent	8 (24.2)	2 (9.5)
	Total	14 (20.3)	3 (6.3)
cefuroxime axetil	Soil	6 (16.7)	1 (3.7)
	Effluent	8 (24.2)	2 (9.5)
	Total	14 (20.3)	3 (6.3)
cefoxitin	Soil	3 (8.3)	1 (3.7)
	Effluent	8 (24.2)	0 (0)
	Total	11 (15.9)	1 (2.1)
cefuroxime	Soil	3 (8.3)	1 (3.7)
	Effluent	6 (18.2)	1 (4.8)
	Total	9 (13.0)	2 (4.2)
ceftazidime	Soil	3 (8.3)	1 (3.7)
	Effluent	6 (18.2)	1 (4.8)
	Total	9 (13.0)	2 (4.2)
ceftriaxone	Soil	3 (8.3)	1 (3.7)
	Effluent	6 (18.2)	1 (4.8)
	Total	9 (13.0)	2 (4.2)

cefepime	Soil	3 (8.3)	1 (3.7)
	Effluent	6 (18.2)	1 (4.8)
	Total	9 (13.0)	2 (4.2)
aztreonam	Soil	3 (8.3)	1 (3.7)
	Effluent	6 (18.2)	1 (4.8)
	Total	9 (13.0)	2 (4.2)
meropenem	Soil	0 (0)	1 (3.7)
	Effluent	1 (1.4)	0 (0)
	Total	1 (1.4)	1 (2.1)
amikacin	Soil	0 (0)	0 (0)
	Effluent	0 (0)	0 (0)
	Total	0 (0)	0 (0)
gentamicin	Soil	7 (19.4)	0 (0)
	Effluent	7 (21.2)	1 (4.8)
	Total	14 (20.3)	1 (2.1)
ciprofloxacin	Soil	12 (33.3)	2 (7.4)
	Effluent	14 (42.4)	3 (14.3)
	Total	26 (37.7)	5 (10.4)
nitrofurantoin	Soil	0 (0)	0 (0)
	Effluent	4 (5.8)	1 (4.8)
	Total	4 (5.8)	1 (2.1)
trimethoprim-sulfamethoxazole	Soil	23 (63.9)	6 (22.2)
	Effluent	22 (66.7)	5 (23.8)
	Total	45 (65.2)	11 (22.9)
